# Supplementary material for: Year-round water management for desert bighorn sheep corresponds with visits by predators not bighorn sheep
Source: PLoS One. 2020 Nov 24;15(11):e0241131. doi: 10.1371/journal.pone.0241131 (PMC7685489; doi:10.1371/journal.pone.0241131)
Supplement: Attachment — Submitted filename: review.pdf [file pone.0241131.s003.pdf]

Abstact

Line 23. Add lowercase to the beginning of the scientific name

Introduction

Line 53. I suggest that you review the article of Escobar Flores et al. 2019 : Waterhole detection using a vegetation index in desert bighorn sheep (*Ovis canadensis cremnobates*) hábitat. The support information of this article mentions a coincidence of the visits of waterholes by bighorn and predators.

Materials and methods

Line 116. Please datial the characteristics of the camera for example: trigger speed, setting that capture per trigger…etc….Examples of this can be found in the following articles:

Perry TW, Newman T, Thibault KM. Evaluation of methods used to estimate size of a population of desert bighorn sheep (*Ovis canadensis mexicana*) in New Mexico. Southw Nat. 2010;55: 517–524.

Whiting, J. C., Bowyer, R. T., Flinders, J. T., Bleich, V. C., & Kie, J. G. (2010). Sexual segregation and use of water by bighorn sheep: implications for conservation. *Animal Conservation*, *13*(6), 541-548.

Escobar-Flores, J. G., Sandoval, S., Valdez, R., Shahriary, E., Torres, J., Alvarez-Cardenas, S., & Gallina-Tessaro, P. (2019). Waterhole detection using a vegetation index in desert bighorn sheep (Ovis canadensis cremnobates) habitat. *PloS one*, *14*(1), e0211202.

Line 174. Please mention with which instrument the temperature, relative humidity and precipitation were measure.
